# Molecular Mechanics Study of the Cyclic and Unsymmetric Diborane(4) Compounds

**DOI:** 10.1100/tsw.2003.48

**Published:** 2003-08-02

**Authors:** N. Rendtorff, E. A. Castro

**Affiliations:** CEQUINOR, Departamento de Química, Facultad de Ciencias Exactas, Universidad Nacional de La Plata, C.C. 962, La Plata 1900, Buenos Aires, Argentina

**Keywords:** Diborane (4) compounds, molecular mechanics, molecular geometry

## Abstract

The results of a theoretical study on the structure of some diborane(4) compounds are presented in order to analyze the issue related to the relative stabilities of the 1,1- vs. 1,2-isomers. Through the employment of the molecular mechanics method, characteristic distances and angles are given and they are compared with available experimental data. In order to rationalize the results, the different energy components are discussed in a comparative fashion. We find a rather satisfactory agreement between theoretical and experimental data. Some possible future extensions are pointed out to complement this sort of analysis.

http://preprint.chemweb.com/physchem/0205003

## INTRODUCTION

Derivatives of diborane(4) are an important class of compound in boron chemistry, part of their interest deriving from the presence of an unsupported two-center, two-electron B-B bond[1]. Knowledge of the chemistry of boron compounds has accelerated rapidly in recent years, and a variety of new boron compounds have been prepared and studied in detail. However, there are several aspects of the chemistry of such species that have been developed only recently.

Among the most relevant molecular features under present discussion, the structural existence of 1,1-isomers and 1,2-isomers poses the natural question about their relative stabilities. Actually, the 1,1- *vs*. 1,2-isomers issue was raised long ago and was first discussed by Shore et al.[2,3] and later by Nöth et al.[4,5]. Recently, some experimental papers have been published dealing with this topic and some preliminary electronic structure calculations have been reported to complement those studies[6,7]. In most cases, 1,1-isomers are the favored structures over the 1,2-isomers, although cyclic 1,2-species can be found. Besides, derivatives of diborane(4) are essentially planar, but there exist structural data for some molecules showing a slight twist around the B-B bond.

This diversity of experimental information is worthy of a systematic theoretical analysis in order to gain some understanding of the basic reasons determining the molecular structure of the different derivatives of diborane(4). This paper deals with a theoretical structural study on the basis of an empirical theoretical procedure of some diborane(4) compounds and it may be considered as a first step in such a direction. The chosen molecular set consists of the following six molecules: **(1)** $B_2(cat)_2$ (cat = 1,2-$O_2C_6H_4$), **(2)** $B_2(1,2\text{-}S_2C_6H_4)_2$, **(3)** 1,2-$B_2(NMe_2)_2$(cat), **(4)** 1,2-$B_2(NMe_2)_2$ (thiocat) (thiocat = 1,2-$S_2C_6H_4$), **(5)** $B_2$(thiocat)(pin) (pin = $(OCH_2)_2$), and **(6)** $B_2$(thiocat)(cat). The following scheme shows the geometrical arrangements of 1,1- and 1,2-isomers of compound **1**.

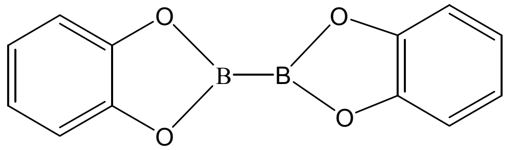

1,2-isomer

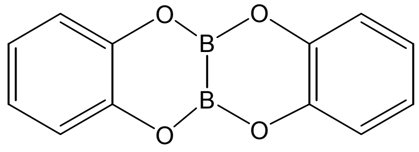

1,1-isomer

**Scheme 1**

## METHOD OF CALCULATION

Many of the problems one would like to tackle in molecular modeling are unfortunately too large to be considered by quantum mechanical methods based on strict first principles. Molecular mechanics methods (also known as force field methods) ignore the electronic motions and calculate the energy of a system as a function of the nuclear positions only. In some cases, force fields can provide answers that are as accurate as even the highest level quantum mechanical calculations, in a fraction of the computer time. Molecular mechanics cannot, of course, provide properties that depend on the electronic distributions in a molecule. That molecular mechanics works at all is due to the validity of several assumptions. The first of these is the Börn-Oppenheimer approximation, without which it would be impossible to contemplate writing the energy as a function of the nuclear coordinates at all.

Molecular mechanics is based on a rather simple model of the interactions within a system with contributions from processes such as the stretching of bonds, the opening and closing of angles, and the rotations about single bonds. Even though simple functions (e.g., Hooke's law) are used to describe these contributions, the force field can perform quite acceptably. The idea that a molecule can be modeled as a collection of balls joined together with springs is of long standing. The molecular mechanics model capitalizes on this idea by seeking to express the intramolecular potential energy as a sum of terms that comprises the differences between actual and reference values geometries[8]. The large number of applications of this method, the interesting results derived for a number of physical-chemistry and structural properties, and its present use in several chemistry areas renders further justifications for its employment unnecessary[9].

We have resorted to the application of the MM+ force field included in the HYPERCHEM® for Windows package[10]. This force field was developed for organic molecules and is an "all atom" force field. The provision of additional parameters (i.e., force constants) through two alternative schemes[11] extends the range of chemical compounds that MM+ can accommodate. We have chosen the Polak-Ribiére (conjugated gradient) minimization algorithm, with a RMS gradient of 0.01 kcal/mol as termination condition. The minimization procedure was run without any restriction and in all cases convergence condition was reached in quite sensible computing times.

## RESULTS AND DISCUSSION

Table 1 displays the most relevant theoretical and experimental data of the six molecules.

**TABLE 1**
**Experimental and Theoretical Bond Distances of Diborane(4) Compounds**

| | | | Bond Length (Angstrom) | | | | | | | | |
|---|---|---|---|---|---|---|---|---|---|---|---|
| **Molecule** | **Isomer** | **Energy (kcal/mol)** | **B-B** | **B-O** | **C-O** | **C-C** | **C-H** | **B-N** | **N-C** | **B-S** | **C-S** |
| **1** | 1,1 | 0.8640 | 1.69 | 1.48 | 1.36 | 1.39 | 1.10 | — | — | — | — |
| | 1,2 | 20.3200 | 1.67 | 1.46 | 1.37 | 1.40 | 1.10 | — | — | — | — |
| **2** | 1,1 | -1.0077 | 1.69 | — | — | 1.40 | 1.10 | — | — | 1.94 | 1.83 |
| | 1,2 | 25.5554 | 1.68 | — | — | 1.40 | 1.10 | — | — | 1.92 | 1.82 |
| **3** | 1,1 | 14.3605 | 1.69 | 1.47 | 1.37 | 1.37 | 1.10 | 1.53 | 1.45 | — | — |
| | 1,2 | 5.1375 | 1.69 | 1.47 | 1.37 | 1.35 | 1.10 | 1.53 | 1.45 | — | — |
| **4** | 1,1 | 3.3755 | 1.69 | — | — | 1.40 | 1.10 | 1.53 | 1.44 | 1.93 | 1.83 |
| | 1,2 | 19.6126 | 1.70 | — | — | 1.40 | 1.10 | 1.53 | 1.45 | 1.93 | 1.82 |
| **5** | 1,1 | 8.9914 | 1.68 | 1.46 | 1.37 | 1.40 | 1.11 | — | — | 1.93 | 1.83 |
| | 1,2 | 26.7193 | 1.68 | 1.46 | 1.37 | 1.40 | 1.11 | — | — | 1.92 | 1.82 |
| **6** | 1,1 | -1.0228 | 1.69 | 1.48 | 1.36 | 1.40 | 1.10 | — | — | 1.93 | 1.83 |
| | 1,2 | 23.0862 | 1.68 | 1.46 | 1.37 | 1.40 | 1.10 | — | — | 1.92 | 1.82 |

| | | | Selected Bond Angles (degree) | | | | |
|---|---|---|---|---|---|---|---|
| **Molecule** | **Isomer** | **Energy (kcal/mol)** | **C-C-O** | **B-B-N** | **B-B-O** | **S-B-B** | **N-B-O** |
| **1** | 1,1 | 0.8640 | 105.4 | — | 126.0 | — | — |
| | 1,2 | 20.3200 | 104.8 | — | 114.8 | — | — |
| **2** | 1,1 | -1.0077 | 127.7 | — | 125.5 | — | — |
| | 1,2 | 25.5554 | 117.4 | — | 116.0 | — | — |
| **3** | 1,1 | 14.3605 | — | 119.7 | — | — | — |
| | 1,2 | 5.1375 | — | 119.7 | — | — | — |
| **4** | 1,1 | 3.3755 | — | 120.2 | — | — | — |
| | 1,2 | 19.6126 | — | 120.2 | — | — | — |
| **5** | 1,1 | 8.9914 | — | — | — | 128.5 | — |
| | 1,2 | 26.7193 | — | — | — | 117.1 | — |
| **6** | 1,1 | -1.0228 | 117.8 | 123.7 | 114.8 | — | 120.9 |
| | 1,2 | 23.0862 | 127.7 | 120.0 | 125.7 | — | — |

Average experimental bond distances (Angstrom): <B-O> = 1.40, <B-S> = 1.0, <C-C> = 1.40, <B-B> = 1.70, <C-S> = 1.76.

Since molecular mechanics total energies represent not an absolute quantity but a sort of deviation with respect to reference equilibrium values, it is not possible to give a direct interpretation to such data. Energy differences between isomers can be interpreted as a measure of relative stabilities. Analysis of present results shows that preferred conformations are relatively higher than nonpreferred ones by a large energy amount. **3** is the only molecule predicted to be a 1,2-isomer, in close agreement with experimental results[6].

The remaining molecules favor the unsymmetrical isomers (i.e., 1,1-isomers), and there is a satisfactory agreement between both sort of results. In fact, compounds **1**, **2,** and **5**, and almost all related species that have been structurally characterized, exist as 1,1 isomers. Besides, the comparison between experimental and theoretical distances and angles reveals that the present theoretical method predicts rather well the experimental available data. B-O, B-S, and C-S bond lengths are rather overestimated, while B-B and C-C bond lengths are predicted in good agreement with average experimental values. Since there is some dispersion among available experimental structural data for diborane(4) compounds, we have made the corresponding comparisons with average experimental bond distances.

## CONCLUSIONS

We have presented theoretical results related to geometrical parameters of the title compounds. The main point addressed in this study was to analyze the relative stabilities of 1,1- vs. 1,2-isomers, because this point is actually under discussion. Some previous studies have pointed out that, in general, 1,1-isomers are more stable than 1,2-isomers. Our findings are in agreement with this feature. Besides, quantitative comparisons among available experimental data and theoretical results for bond distances are quite satisfactory.

A more complete analysis based on molecular orbital theory or/and density functional theory will give a more complete and comprehensive perspective on the nature of chemical bonding for the title compounds. Work along this line is actually under development and results will be presented elsewhere.
